# Deciphering Important Odorants in a Spirulina (*Arthrospira platensis*) Dietary Supplement by Aroma Extract Dilution Analysis Using Offline and Online Fractionation Approaches

**DOI:** 10.3390/ijms26146767

**Published:** 2025-07-15

**Authors:** Aikaterina Paraskevopoulou, Veronika Mall, Theodoros M. Triantis, Triantafyllos Kaloudis, Anastasia Hiskia, Dimitra Dimotikali, Martin Steinhaus

**Affiliations:** 1 Leibniz Institute for Food Systems Biology at the Technical University of Munich, 85354 Freising, Germany; k.paraskevopoulou@inn.demokritos.gr (A.P.); v.mall.leibniz-lsb@tum.de (V.M.); 2 School of Chemical Engineering, National Technical University of Athens, 15780 Athens, Greece; demot@chemeng.ntua.gr; 3 Institute of Nanoscience and Nanotechnology, National Center for Scientific Research “Demokritos”, 15341 Athens, Greece; t.triantis@inn.demokritos.gr (T.M.T.); kaloudis@eydap.gr (T.K.); a.hiskia@inn.demokritos.gr (A.H.); 4 AquOmixLab, Department of Water Quality Control, Athens Water Supply and Sewerage Company (EYDAP SA), 11146 Athens, Greece

**Keywords:** cyanobacteria, *Arthrospira platensis*, spirulina, food supplement, odorant, gas chromatography–olfactometry (GC–O), aroma extract dilution analysis (AEDA), offline and online fractionation, odorant structure assignment

## Abstract

Investigating the volatiles isolated from a commercial spirulina (*Arthrospira platensis*) dietary supplement by gas chromatography–olfactometry (GC–O) in combination with an aroma extract dilution analysis (AEDA) resulted in 29 odor events with flavor dilution (FD) factors between 8 and 2048. Identification experiments, including various offline and online fractionation approaches, led to the structure assignment of 30 odorants, among which the most potent were sweaty 2- and 3-methylbutanoic acid (FD 2048), roasty, earthy, shrimp-like 2-ethyl-3,5-dimethylpyrazine (FD 2048), vinegar-like acetic acid (FD 1024), and floral, violet-like β-ionone (FD 1024). Static headspace dilution analysis revealed sulfuric, cabbage-like methanethiol (FD factor ≥ 32) as an additional potent odorant. In summary, 31 important spirulina odorants were identified in this study, and 14 were reported for the first time as spirulina constituents. Our data will provide a basis for future odor optimization of spirulina-based food products.

## 1. Introduction

A key global trend in modern food industries, driven by sustainability and cost efficiency, is the pursuit of alternative sources of natural compounds that benefit human health [1]. In this context, cyanobacteria constitute a promising source of biologically active molecules [2]. The genus *Arthrospira platensis* is commercially merchandized under the name spirulina as a dietary supplement rich in nutrients, such as proteins, vitamins, and pigments [1,3]. These chemical groups have been associated with spirulina’s antioxidant, anticancer, anti-inflammatory, and antibacterial activity [4,5]. Various spirulina products, such as dried spirulina biomass powder pressed in flakes and tablets, and convenience food products enriched with bioactive spirulina isolates, can be found in the global market [3].

Despite the advertised high nutritional value of spirulina, its undesirable odor negatively affects consumers’ acceptance [6]. The variation of spirulina cultivation conditions, as well as the processing and storage of spirulina products, affect the composition, and thus the odor profile, of spirulina [6]. In an attempt to understand spirulina odor, previous studies linked spirulina’s volatile profile to the product’s overall odor [7,8,9]. Based on headspace–solid phase microextraction–gas chromatography–mass spectrometry (SPME–GC–MS) analysis, Bao et al. (2018) suggested that 2,5-dimethylpyrazine, 2-methylpyrazine, hexan-1-ol, 3,3,5-trimethylcyclohexan-1-ol, 2,2,6 trimethylcyclohexan-1-one, and 3,5,5-trimethylcyclohex-2-en-1-one contribute to spirulina odor [10]. Other semi-quantitative studies on spirulina volatiles using SPME–GC–MS suggested 2-methylpropanal, hexadecanamide, and hexadecanoic acid derivatives as potentially crucial odorants [7]. However, none of these studies applied activity-guided odorant screening approaches, and thus the correlations found between volatile compounds and overall odor may not have a causal background [7,9,10,11]. SPME sampling involves additional risks. For example, competitive effects during adsorption may suppress trace odorants, and the high temperature during desorption may lead to thermal artifact formation [12,13,14,15,16].

In general, odorants are volatile compounds that bind to and activate at least one of the ~400 human olfactory receptors [17]. Key food odorants (KFOs) are odorants that are present in a food in concentrations above their odor threshold concentration and verifiably contribute to the overall odor [18]. To unravel the odor of spirulina products on a molecular level, i.e., to identify the causal odor-active volatiles, gas chromatography–olfactometry (GC–O) is indispensable. The human olfactory system is utilized in GCO as a selective and sensitive detector for odor-active compounds [18]. A first odor potency assessment of the odorants is possible by applying aroma extract dilution analysis (AEDA) [19]. Comparing olfactory, chromatographic, and mass spectral data with those of authentic reference compounds leads to unequivocal structure assignments [18]. Allegedly time-saving methods, such as directly combining GC–O with mass spectrometry (GC–O/MS) for quick structure assignment, pose a high risk of misidentification due to coelution. However, elaborated offline pre-fractionation of the volatile isolate and/or online fractionation, e.g., by two-dimensional gas chromatography (GC) approaches, are suitable tools to avoid such coelution problems and substantially increase the success in structure elucidation, especially of trace odorants [12,20,21].

Recently, Jia et al. (2024) [22] combined two-dimensional comprehensive gas chromatography–mass spectrometry (GC × GC–MS) and GC–tandem mass spectrometry (MS/MS) data with GC–O data and reported 37 odorants in dried spirulina powder. Potent odorants were hexanal, oct-1-en-3-ol, β-cyclocitral (2,6,6-trimethylcyclohexene-1-carbaldehyde), and β-ionone ((3*E*)-4-(2,6,6-trimethylcyclohex-1-en-1-yl)but-3-en-2-one). Nevertheless, the literature on spirulina odorants is scarce.

This investigation sought to locate the odorants present in a volatile isolate of a spirulina dietary supplement using GC–O and to assess their odor potency through AEDA. Offline and online fractionation approaches, including two-dimensional heart-cut gas chromatography–olfactometry/mass spectrometry (GC–GC–O/MS) and comprehensive GC×GC–MS, were employed to facilitate structure elucidation. Highly volatile odorants were addressed by static headspace (SH)–GC–O/MS and the application of SH dilution analysis [23].

## 2. Results and Discussion

### 2.1. Odorants in the Spirulina Dietary Supplement

Following common practice, i.e., spirulina flakes or powder marketed as dietary supplements being typically stirred into beverages before consumption, we added water before solvent extraction. This procedure, in addition, produced extracts with a more intense odor than a water-free workup—an observation that was in line with previous reports on the liberation of odorants from low-moisture foods upon water contact [24,25]. Furthermore, diethyl ether was used as the extraction solvent because the extract, when evaluated on a strip of filter paper after evaporation of the solvent, was rated more similar in odor to the starting material than an extract obtained with dichloromethane.

**Figure 1:**
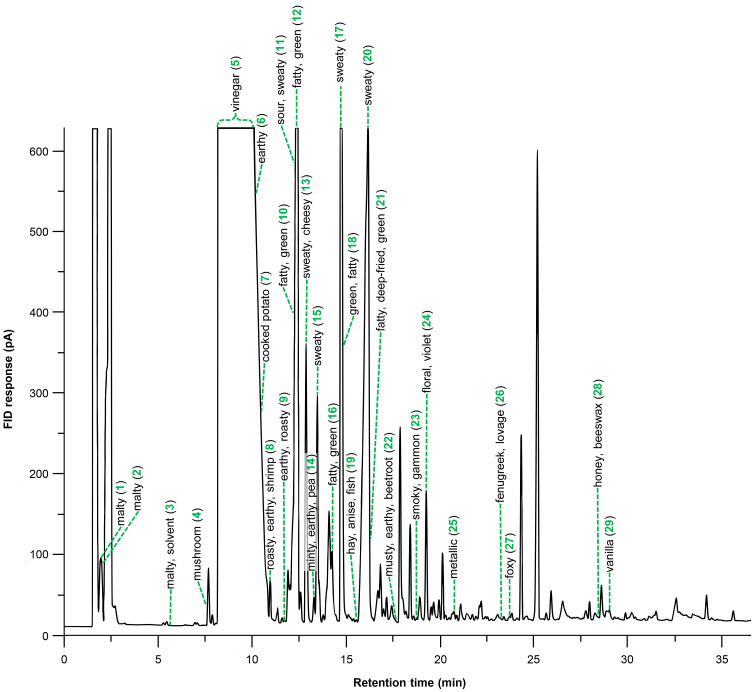
Flame ionization detector (FID) chromatogram obtained by gas chromatography–olfactometry (GC–O) of the volatile isolate obtained from the spirulina dietary supplement (odor events with flavor dilution (FD) factors ≥ 8). The detected odor events are numbered in ascending order.

The gas chromatography–olfactometry (GC–O) investigation of the spirulina volatile isolate obtained after solvent extraction, automated solvent-assisted flavor evaporation (aSAFE), and concentration revealed 29 distinct odor events (Figure 1). Some odor events were linked with prominent peaks in the flame ionization detector (FID) chromatogram, such as **13** (sweaty, cheesy), **15**, and **20** (both sweaty). Other prominent FID peaks included two or more odor events. For example, the most prominent peak eluted in the retention time window of 8.1–11.3 min and included three distinct odor events, namely **5** (vinegar-like), **6** (earthy), and **7** (cooked potato-like). However, smaller peaks also covered more than one odor event, e.g., the peak at 12.4 min included **10** (fatty, green), **11** (sour, sweaty), and **12** (fatty, green), and the peak at 14.9 min included the events **17** (sweaty) and **18** (green, fatty). Furthermore, there were also odorant events in the chromatogram with very low FID response, indicating potent odorants in trace concentrations, e.g., **3** (malty, solvent-like), **19** (hay-like, anise-like, fishy), **25** (metallic), **26** (fenugreek-like, lovage-like), and **28** (honey-like, beeswax-like). In contrast, some of the more prominent peaks showed no odor at all, e.g., those at 18.1, 24.6, and 25.5 min. Among them might be alkanes, which were recently identified as major volatiles in spirulina [26]. In summary, the GC–O data demonstrated the need for further fractionation of the volatile isolate before gas chromatography–mass spectrometry (GC–MS) analysis could be expected to be helpful for the structure assignment of the odorants.

**Figure 2:**
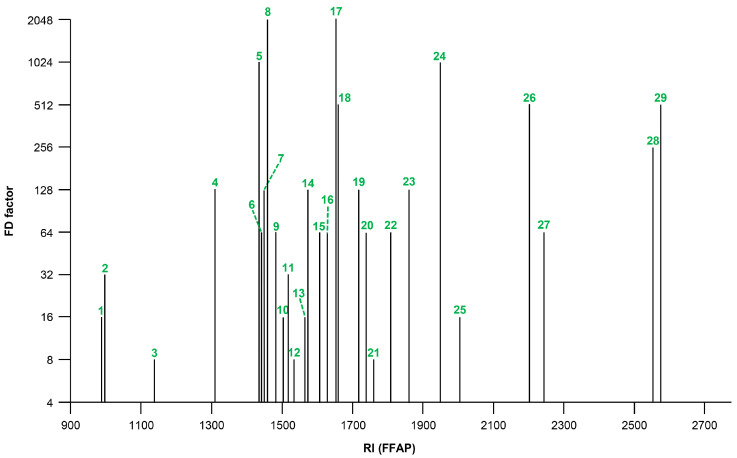
Flavor dilution (FD) chromatogram obtained by applying aroma extract dilution analysis (AEDA) to a volatile isolate of the spirulina dietary supplement (odor events with FD factors ≥ 8); FD factors plotted against retention indices (RIs). The numbering is aligned with that of Figure 1.

Before we started with the structural elucidation, we carried out an aroma extract dilution analysis (AEDA) to gain an initial insight into the odor potency of the individual odor events. This approach revealed flavor dilution (FD) factors for the 29 odor events, ranging from 8 to 2048. The events eliciting roasty, earthy, shrimp-like (**8**) and sweaty (**17**) odors were found to have the highest FD factor of 2048. Somewhat lower FD factors were determined for the odor events **5** (vinegar-like; FD 1024), **24** (floral, violet-like; FD 1024), **18** (fatty, green; FD 512), **26** (fenugreek-like, lovage-like; FD 512), and **29** (vanilla-like, sweet; FD 512). An FD chromatogram plotting the FD factors of the major odor events against the respective retention indices (RI) on the DB-FFAP column is depicted in Figure 2.

Comparing the odor quality and the respective RI obtained for each odor event by using two GC columns of different polarity (DB-FFAP and DB-5) with data available in the open-access Leibniz-LSB@TUM Odorant Database (https://www.leibniz-lsb.de/en/databases/leibniz-lsbtum-odorant-database; accessed on 5 May 2025) [27] provided initial indications of possible underlying odorant structures. As expected, confirmation of the structure proposals by mass spectrometry was challenging.

In most cases, such as for the structure assignment of the odorants **3**–**5**, **10**–**13**, **15**–**23**, **28**, and **29** in the spirulina volatile isolate, two-dimensional comprehensive gas chromatography–time-of-flight–mass spectrometry (GC×GC–TOFMS) analyses provided sufficient separation to obtain clear mass spectra. Hence, no further off-line fractionation was necessary for the structure elucidation of, e.g., malty solvent-like butan-1-ol (**3**), vinegar-like acetic acid (**5**), fatty, green (2*E*)-non-2-enal (**12**), honey, beeswax-like phenylacetic acid (**28**), and vanilla-like, sweet vanillin (4-hydroxy-3-methoxybenzaldehyde; **29**). Despite the coelution of the two sweaty-smelling skeletal isomers of methylbutanoic acid (**17a** and **b**) during GC–O analysis, the presence of both 2-methylbutanoic acid (**17a**) and 3-methylbutanoic acid (**17b**) was confirmed by investigating both authentic reference compounds and the sample using GC×GC–TOFMS, resulting in two separated blobs with the respective characteristic mass spectrum (EI).

Preceding offline fractionation was necessary to overcome mass spectral interferences derived from coeluting matrix components in the unequivocal identification of odorants **6**–**9**, **14**, and **26**. These experiments involved the initial separation of neutral/basic (NBV) and acidic volatiles (AV) by liquid–liquid extraction and further fractionation of the NBV fraction into five subfractions of different polarity by liquid chromatography. All fractions and subfractions were first screened for the target odorants by GC–O. Analysis of the NBV fraction using GC×GC–TOFMS led to the unequivocal structure assignment of earthy 2,3-diethylpyrazine (**6**) and cooked potato-like 3-methylsulfanylpropanal (**7**). This was particularly facilitated by the fact that acetic acid (**5**), a major coeluting compound in the total volatile isolate, had been separated from the NBV fraction. Further experiments used the NBV fraction and its subfractions in combination with two-dimensional heart-cut gas chromatography–olfactometry/mass spectrometry (GC–GC–O/MS) to identify odorants **8**, **9**, **14**, and **24**. After identifying the target odorant in the GC–O chromatogram of the first dimension, the respective eluate portion of a subsequent run was transferred via a Deans switch to a second GC column of different polarity and re-chromatographed. This procedure enabled compounds coeluting in the first dimension to be separated in the second dimension, allowing for the detection of clear mass spectra in electron ionization (EI) and chemical ionization (CI) mode and, simultaneously, the pristine odor perception at the sniffing port. Following this approach, earthy, roasty-smelling 2,3-diethyl-5-methylpyrazine (**9**) and earthy, pea-like 2-butyl-3-methylpyrazine (**14**) were successfully identified in subfraction NBV3. Floral, violet-like smelling β-ionone ((3*E*)-4-(2,6,6-trimethylcyclohex-1-en-1-yl)but-3-en-2-one **24**) was successfully identified in the NBV fraction.

Analyzing subfraction NBV3 by GC–GC–O/MS resulted in clear mass spectra in EI and CI mode for roasty, earthy, shrimp-like smelling odorant **8**. Data indicated a molecular ion of *m*/*z* 136. Matching the EI spectrum with the NIST library 2.3 (2017) suggested 3-ethyl-2,5-dimethylpyrazine as the structure. However, the GC–O analysis of the authentic reference compound using the GC–O/FID instrument resulted in a different odor quality and different RI values, thus disqualifying this structure proposal. The unquestionable similarities in the mass spectra led us to test positional isomers of 3-ethyl-2,5-dimethylpyrazine. GC–O and GC–GC–O/MS data identical to odorant **8** were obtained from 2-ethyl-3,5-dimethylpyrazine (Table 1). Thus, roasty, earthy, shrimp-like odorant **8** was finally identified as 2-ethyl-3,5-dimethylpyrazine.

Due to the particular sensitivity and selectivity of negative chemical ionization (NCI) towards epoxy derivatives, metallic-smelling trace compound **25** was unequivocally identified as *trans*-4,5-epoxy-(2*Ε*)-dec-2-enal ((2*E*)-3-[2*R*,3*R*)/(2*S*,3*S*)-3-pentyloxiran-2-yl]prop-2-enal; **25**) by analyzing the spirulina volatile isolate and the authentic reference compound with the GC–GC–O/MS instrument in NCI.

In summary, analyzing authentic reference compounds by GC–O and GC–MS under the same conditions as applied to the spirulina volatile isolate and comparing the obtained data, i.e., RI, odor quality, and mass spectra, led to the unequivocal identification of 26 out of 30 spirulina odorants detected during GC–O (Table 2). However, to obtain pure mass spectra of the spirulina odorants and avoid interferences of coeluting compounds, elaborate fractionation of the spirulina volatile isolate by different off-line and on-line fractionation approaches has been proven to be necessary. Finally, we could not obtain mass spectra for two trace odorants, despite our best efforts. These two compounds were fenugreek-like, lovage-like **26**, and foxy **27**. Based on RI and olfactory data (odor quality and intensity), they could, however, be identified as sotolon (3-hydroxy-4,5-dimethylfuran-2(5*H*)-one; **26**) and 1-(2-aminophenyl)ethanone (**27**).

The workup for AEDA, especially the concentration step involving Vigreux columns to distill off the bulk of the solvent, bears the risk of losing very highly volatile compounds; especially those with boiling points lower than that of the extraction solvent will evaporate. Thus, static headspace (SH)–GC–O in combination with SH dilution analysis was carried out as a complementary screening approach to GC–O and AEDA to cover highly volatile odorants [18]. As a result, only a single additional odorant was detected in the static headspace above a spirulina suspension, namely sulfuric, cabbage-like methanethiol, for which an FD factor of ≥32 was calculated. SH–GC–O/MS additionally confirmed the structure assignments of the early eluting odorants 3- and 2-methylbutanal previously detected as odorants **1** and **2** in the AEDA, as the solvent-free sample preparation facilitated recording of clean mass spectra.

### 2.2. Discussion

In summary, 30 odor events were detected by applying AEDA and SH dilution analysis. By subjecting the volatile isolate of the spirulina suspension to various offline and online fractionation approaches and subsequent mass spectrometry, 26 odorant structures were unequivocally assigned. The odorant structure assignment of three additional compounds was accomplished by investigating the static headspace above the spirulina suspension. Two structure assignments were based on RI and olfactory data only, since mass spectra could not be obtained due to the trace amounts of the odorants. In total, 31 odorant structures were deciphered in the investigated spirulina dietary supplement.

**Figure 3:**
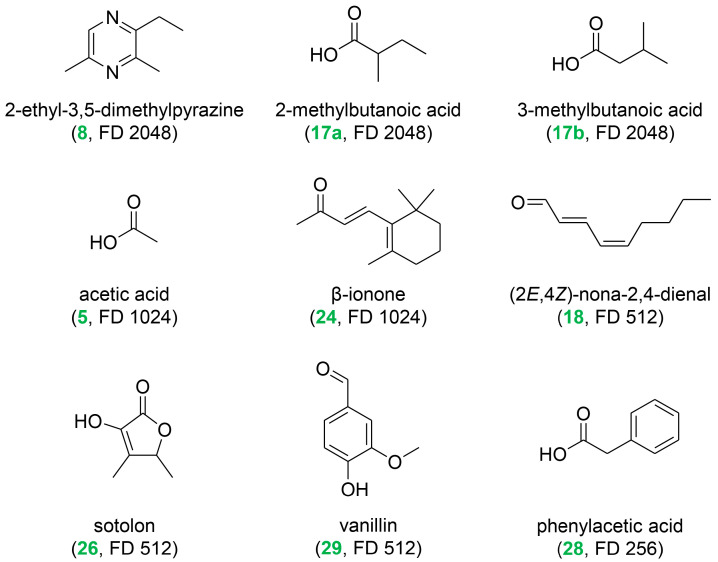
Structures of the major odorants identified in the spirulina dietary supplement (numbering refers to Table 2; flavor dilution (FD) factors in parentheses).

The highest FD factors (≥256) were determined for the nine odorants displayed in Figure 3. Among them, roasty, earthy, shrimp-like 2-ethyl-3,5-dimethylpyrazine (**8**) and the sum of sweaty 2- and 3-methylbutanoic acid (**17a** and **b**) showed the highest FD factor of 2048. Floral, violet-like β-ionone (**24**) and vinegar-like acetic acid (**5**) were identified with an FD factor of 1024. Somewhat lower FD factors of 512 and 256 were determined for green, fatty (2*E*,4*Z*)-nona-2,4-dienal (**18**), fenugreek-like, lovage-like sotolon (**26**), vanilla-like, sweet vanillin (**29**), and phenylacetic acid (**28**), eliciting a honey-like, beeswax-like scent. This investigation identified 14 compounds (**3**, **6**, **8**–**10**, **13**–**14**, **18**–**19**, **23**, **26**–**29**) in spirulina for the first time. It is worth mentioning that eight of these novel constituents showed high FD factors ≥128 and hence may be important contributors to the overall olfactory profile of the spirulina dietary supplement.

2- and 3-methylbutanoic acid (**17a** and **b**) have been previously identified in spirulina, although they were considered to be of minor importance [22]. Nevertheless, the two isomers of methylbutanoic acid have been detected as key food odorants (KFOs) in many food products [33]. They derive from the amino acids isoleucine and leucine, respectively, both of which are present in high concentrations in spirulina [34,35]. Vinegar-like acetic acid (**5**), emerging among the major odorants (FD 1024), has been previously reported as volatile in spirulina and other microalgae [22,29,32]. Other odorant carboxylic acids present in the spirulina dietary supplement were phenylacetic acid (**28**) and propanoic acid (**11**) (FD 256 and 32, respectively). Phenylacetic acid (**28**), formed, e.g., by the Strecker reaction from phenylalanine, another amino acid present in spirulina [36,37], was found for the first time in spirulina. Propanoic acid (**11**) has previously been reported as a spirulina volatile compound [10].

Based on its high FD factor of 2048, roasty, earthy, shrimp-like 2-ethyl-3,5-dimethylpyrazine (**8**) was rated as one of the most important spirulina odorants. This pyrazine has previously been reported in the volatile profile of the microalga *Tetraselmis* and is also known as a key odorant in fish soup [7,38]. In earlier investigations on the volatile profile of spirulina, 2-ethyl-3,5-dimethylpyrazine was not mentioned, but its isomers 5-ethyl-2,3-dimethylpyrazine and 3-ethyl-2,5-dimethylpyrazine were reported [9,22,39]. Given the three isomers’ similar retention behavior and mass spectra, earlier studies might have misidentified them. Other odorant pyrazines found with slightly lower FD factors, such as 2,3-diethylpyrazine (**6**), 2,3-diethyl-5-methylpyrazine (**9**), and 2-butyl-3-methylpyrazine (**14**), were found for the first time in spirulina. The earthy, roasty 2,3-diethyl-5-methylpyrazine (**9**) is among the most frequent pyrazines found as a KFO in thermally treated foods [33]. Pyrazines are generally formed during thermal processing by the Maillard reaction, strongly impacting the sensory properties of such food products [40]. High temperatures during the drying process of the fresh spirulina biomass may explain the formation of these odorants.

Floral, violet-like β-ionone (**24**; FD 1024), which naturally occurs in foods rich in β-carotene, has been reported in spirulina and other microalgae species, such as *Rhodomonas* and *Tetraselmis* [7,8,9,11,22,26,32]. Based on a high FD factor and the comparison of semi-quantitative data with its odor threshold concentration, this odorant was previously suggested as one of the most important spirulina odorants [22].

(2*E*,4*Z*)-Nona-2,4-dienal (**18**), sotolon (**26**), and vanillin (**29**) were reported for the first time as spirulina constituents in this study. The oxidative degradation of lipids forms the green, fatty (2*E*,4*Z*)-nona-2,4-dienal (**18**) [41,42]. This aldehyde has been described as an important odorant in other microalgae, such as *Crypthecodinium cohnii*, *Chlorella vulgaris*, and *Schizochytrium limacinum* [43]. Sotolon (**26**), with a fenugreek-like, lovage-like odor, is formed biochemically or thermally by the Maillard reaction [44]. Vanilla-like-smelling vanillin (**29**) typically derives from the thermal degradation of chlorogenic acid [45]. Both sotolon (**26**) and vanillin (**29**) belong to the group of “generalists,” affecting the overall olfactory profile of a vast variety of food products, e.g., green tea, prawn meat, and orange juice [33].

The novel spirulina odorant oct-1-en-3-one (**4**) is another lipid oxidation product and is formed from linoleic acid [41,46]. This odorant has the same mushroom-like odor as the previously mentioned spirulina volatile oct-1-en-3-ol [7,9,22,26]. Based on semiquantitative estimations, this alcohol was suggested to contribute to the overall olfactory profile of microalgae [7,32]. Although oct-1-en-3-one and oct-1-en-3-ol elicit the same mushroom-like odor, they substantially differ in their odor threshold concentrations: that of the ketone is 2800 times lower than that of the alcohol [27]. Their distinction can be challenging since they feature similar RI values on a nonpolar GC column. Considering the different odor potency, a coelution of oct-1-en-3-one and oct-1-en-3-ol might quickly lead to a false identification of oct-1-en-3-ol as the mushroom odor-causing compound.

3-Methylsulfanylpropanal (**7**; FD 128), with its characteristic smell of cooked potato, is a Strecker aldehyde derived from methionine and was previously identified among the volatiles of spirulina and other microalgae [9,22,32,36]. Another methionine degradation product and important spirulina odorant candidate is methanethiol, which was detected during the SH dilution analysis. Together with the Strecker aldehydes 3- and 2-methylbutanal (**1** and **2**), methanethiol has been reported earlier in spirulina [9,22,26,47].

While 3-methylnonane-2,4-dione (**19**) and 2-methoxyphenol (**23**) have been identified in spirulina for the first time in this study, earthy, beetroot-like geosmin ((4*S*,4a*S*,8a*R*)-4,8a-dimethyloctahydronaphthalen-4a(2*H*)-ol) (**22**) has been widely reported in studies on the impact of cyanobacteria contamination on off-flavors in drinking water [48,49]. This terpenoid is known to be produced by various cyanobacteria species. However, only a limited number of previous studies reported this potent trace odorant in spirulina [30,31].

## 3. Materials and Methods

### 3.1. Dried Spirulina Flakes

This was a dietary supplement product available on the Greek retail market. According to the manufacturer, *Arthrospira platensis* was cultivated in raceway ponds under greenhouse conditions. The raw biomass was naturally dried. The product was stored according to the label instructions in a cool and dry environment and analyzed before the indicated expiration date.

### 3.2. Chemicals

The reference odorants **1**–**3** and **27** were purchased from Thermo Fisher Scientific (Waltham, MA, USA). Odorants **6**–**9**, **11**–**13**, **15**–**18**, **20**–**24**, **26**, **28**, and **29** were obtained from Merck (Darmstadt, Germany). Odorant **19** was purchased from Chemos (Altdorf, Germany).

The reference odorants methanethiol, (3*Z*,6*Z*)-nona-3,6-dienal (**10**), 2-butyl-3-methylpyrazine (**14**), and *trans*-4,5-epoxy-(2*E*)-dec-2-enal (**25**) were prepared according to procedures reported previously [50,51,52,53].

Dichloromethane, diethyl ether, and *n*-pentane were purchased from CLN (Langenbach, Germany). Before use, the solvents were freshly distilled through a column (120 cm × 5 cm) packed with Raschig rings.

*n*-Hexane, hydrochloric acid (32%), and anhydrous sodium sulfate were purchased from Merck. Sodium hydrogen carbonate (99%) was obtained from Alfa Aesar (Thermo Fisher Scientific; Waltham, MA, USA). Silica gel 60 (0.040–0.63 mm) was obtained from VWR (Darmstadt, Germany) and purified with hydrochloric acid.

### 3.3. Isolation of Spirulina Volatiles

Dried spirulina flakes (2.5 g) were pulverized and dispensed in 6 mL water. The mixture was stirred with diethyl ether (250 mL) at room temperature in the dark for 2 h. The aqueous phase was separated, and extraction was repeated with another portion of diethyl ether (250 mL). The organic layers were combined and subjected to automated solvent-assisted flavor evaporation (aSAFE) [15] at 40 °C and a combination of 0.2 s and 10 s for the open and closed periods of the pneumatic valve. A high vacuum (10^−4^–10^−5^ mbar) was maintained using a high vacuum pump system PT50 (Leybold-Heraeus, Cologne, Germany). The SAFE distillate was dried with anhydrous sodium sulfate and concentrated using a Vigreux column (50 × 1 cm) and, for volumes < 1 mL, a microdistillation device at a water bath temperature of 40 °C [54].

### 3.4. Offline Fractionation

#### 3.4.1. Separation of Neutral/Basic Volatiles (NBV) and Acidic Volatiles (AV)

The dried SAFE distillate was concentrated to approximately 50 mL using a Vigreux column (50 × 1 cm) and vigorously shaken with an aqueous sodium hydrogen carbonate solution (0.5 mol/L; 3 × 50 mL). The organic phase containing NBV was washed with brine (3 × 50 mL) and dried over anhydrous sodium sulfate. The aqueous phases were combined (150 mL), the pH was adjusted to 2.5 using hydrochloric acid (1 mol/L), and the protonated acids were re-extracted using diethyl ether (3 × 100 mL). The combined diethyl ether phases containing AV were dried over anhydrous sodium sulfate. AV and NBV fractions were concentrated to 1 mL using a Vigreux column (50 × 1 cm), and further to 0.1 mL using a microdistillation device.

#### 3.4.2. Fractionation of NBV by Liquid Chromatography

Hexane (1 mL) was added to the concentrated NBV fraction (1 mL), and the mixture was re-concentrated to 1 mL using a Vigreux column (50 × 1 cm). A water-cooled (13 °C) glass column (30 × 1 cm) sealed with degreased cotton wool and sea sand was packed with purified silica gel 60 (0.063–0.2 mm, 7% water; 9 g), and conditioned with pentane (100 mL). NBVs were applied onto the column, and their elution was performed with pentane (100 mL) followed by pentane/diethyl ether mixtures (90 mL + 10 mL, 70 mL + 30 mL, 50 mL + 50 mL), and diethyl ether (100 mL). The eluate was collected in five portions of 100 mL each, and the portions were concentrated to 0.1 mL (fractions NBV1–NBV5). All volatile isolates were stored at −20 °C before analysis.

### 3.5. Gas Chromatography (GC)

Gas chromatography–olfactometry (GC–O) analyses were performed by using a gas chromatograph equipped with a cold-on-column inlet, a custom-made sniffing port, and a flame ionization detector (FID).

For the purpose of identification, experiments were conducted using a two-dimensional comprehensive gas chromatography–mass spectrometry (GC×GC–MS) instrument equipped with a time-of-flight (TOF) MS and a two-dimensional heart-cut gas chromatography–olfactometry/mass spectrometry (GC–GC–O/MS) instrument equipped with an orbitrap mass spectrometer. Highly volatile odorants were analyzed using a static headspace (SH)–GC–O/MS instrument equipped with an ion trap MS. Detailed specifications of all used GC instruments are provided in the Supplementary Materials.

### 3.6. Aroma Extract Dilution Analysis (AEDA)

A concentrated spirulina volatile isolate (0.1 mL) was stepwise diluted with diethyl ether at a ratio of 1:2 (*v*/*v*) to obtain dilutions of 1:2, 1:4, 1:8, 1:16, …, 1:2048. Each diluted sample was analyzed by GC–O using the GC–O/FID system with the DB-FFAP column. Two assessors (both female, aged 32–39) conducted the analyses. A flavor dilution (FD) factor was assigned to each located odor event representing the dilution factor of the highest diluted sample, in which the odor event was detected during GC–O by either of the two assessors.

### 3.7. SH Dilution Analysis

Dried spirulina flakes (1 g) were pulverized and dispensed in 3 mL of water. The mixture was placed in a 120 mL vial, which was sealed gastight. After stirring the mixture for 15 min at room temperature in the dark, volumes of 0.125–10 mL headspace were withdrawn from the vial with a tempered (40 °C) gastight syringe and injected into the SH–GC–O/MS instrument. SH dilution analysis was performed by analyzing a series of halved headspace volumes (10 mL, 5 mL, 2.5 mL, 1.25 mL, 0.5 mL, 0.25 mL) using SH–GC–O. An FD factor was assigned to each odorant representing the initial headspace volume divided by the lowest volume in which the odorant was detected during SH–GC–O.

## 4. Conclusions

In conclusion, this study successfully elucidated the important odorant structures in a commercial spirulina dietary supplement by subjecting the volatile isolate of the spirulina suspension to gas chromatography–olfactometry and various offline and online fractionation approaches with subsequent mass spectrometry, as well as by the analysis of the headspace above the spirulina suspension. Subsequent quantitative studies, calculation of odor activity values, and sensory experiments, such as reconstitution and omission tests, will clarify the actual contribution of individual spirulina odorants to the overall olfactory profile and to the hedonic rating of the dietary supplement. This will provide a basis for future studies, e.g., on the impact of strain selection and technological process parameters (drying method, temperature, and time; storage temperature and time etc.), ultimately leading to optimized spirulina food products with tailored and more desirable odor properties.

## Figures and Tables

**Table 1:** Odor qualities, retention indices, and mass spectra (EI) of odorant **8** and two ethyldimethylpyrazines.

Odorant	Odor Quality ^1^	RI ^2^		MS (EI) ^3^: *m*/*z* (%)
		DB-FFAP	DB-5	
odorant **8**	roasty, earthy, shrimp	1450	1089	39 (76), 40 (15), 41 (18), 42 (70), 52 (28), 53 (40), 56 (29), 108 (18), 135 (100), 136 (59)
3-ethyl-2,5-dimethylpyrazine	nutty, earthy	1442	1083	39 (83), 40 (29), 41 (24), 42 (100), 53 (25), 56 (41), 107 (29), 108 (24), 135 (96), 136 (64)
2-ethyl-3,5-dimethylpyrazine	roasty, earthy, shrimp	1450	1089	39 (95), 40 (32), 41 (28), 42 (75), 52 (20), 53 (24), 56 (88), 108 (23), 135 (100), 136 (69)

^1^ Perceived odor quality at the sniffing port during two-dimensional heart-cut gas chromatography–olfactometry (GC–GC–O) analysis. ^2^ Retention index; calculated by linear interpolation from the retention time of the compound and the retention times of adjacent *n*-alkanes. ^3^ Mass spectrum obtained in electron ionization (EI) mode with the two-dimensional heart-cut gas chromatography–olfactometry/mass spectrometry (GC–GC–O/MS) instrument.

**Table 2:** Major odorants (FD factor ≥ 8) in the spirulina volatile isolate.

No.	Odorant ^1^	Odor Quality ^2^	Fraction ^3^	RI ^4^		FD Factor ^5^	Earlier Identified in Spirulina
				DB-FFAP	DB-5		
**1**	3-methylbutanal	malty	NBV	945	665	16	[26,28]
**2**	2-methylbutanal	malty	NBV	953	675	32	[9]
**3**	butan-1-ol	malty, solvent	NBV	1138	667	8	
**4**	oct-1-en-3-one	mushroom	NBV	1305	988	128	[22]
**5**	acetic acid	vinegar	AV	1441	<600	1024	[22,29]
**6**	2,3-diethylpyrazine	earthy	NBV	1443	1085	64	
**7**	3-methylsulfanylpropanal	cooked potato	NBV	1445	1011	128	[9,22]
**8**	2-ethyl-3,5-dimethylpyrazine	roasty, earthy, shrimp	NBV	1450	1089	2048	
**9**	2,3-diethyl-5-methylpyrazine	earthy, roasty	NBV	1482	1154	64	
**10**	(3*Z*,6*Z*)-nona-3,6-dienal	fatty, green	NBV	1500	1096	16	
**11**	propanoic acid	sour, sweaty	AV	1519	750	32	[10]
**12**	(2*Ε*)-non-2-enal	fatty, green	NBV	1532	1169	8	[22]
**13**	2-methylpropanoic acid	sweaty, cheesy	AV	1559	800	16	
**14**	2-butyl-3-methylpyrazine	earthy, pea	NBV	1571	1188	128	
**15**	butanoic acid	sweaty	AV	1616	839	64	[22]
**16**	(2*E*)-dec-2-enal	fatty, green	NBV	1635	1284	64	[22]
**17**	2- and 3-methylbutanoic acid ^6^	sweaty	AV	1660	887	2048	[22]
**18**	(2*E*,4*Z*)-nona-2,4-dienal	green, fatty	NBV	1665	1196	512	
**19**	3-methylnonane-2,4-dione	hay, anise, fishy	NBV	1711	1242	128	
**20**	pentanoic acid	sweaty	AV	1730	941	64	[22]
**21**	(2*E*,4*Z*)-deca-2,4-dienal	fatty, deep-fried, green	NBV	1745	1292	8	[22]
**22**	geosmin	musty, earthy, beetroot	NBV	1816	1413	64	[30,31]
**23**	2-methoxyphenol	smoky, gammon	NBV	1868	1096	128	
**24**	β-ionone	floral, violet	NBV	1944	1491	1024	[7,8,9,11,22,26,32]
**25**	*trans*-4,5-epoxy-(2*E*)-dec-2-enal	metallic	NBV	2012	1375	16	[22]
**26**	sotolon ^7^	fenugreek, lovage	AV	2206	1110	512	
**27**	1-(2-aminophenyl)ethenone ^7^	foxy	NBV	2233	1300	64	
**28**	phenylacetic acid	honey, beeswax	AV	2540	1256	256	
**29**	vanillin	vanilla, sweet	AV	2567	1400	512	

^1^ Odorants exhibiting a flavor dilution (FD) factor of ≥8; if not noted otherwise, structure assignments were based on retention indices (RIs) on two columns of different polarity (DB-FFAP, DB-5), mass spectra obtained by gas chromatography–mass spectrometry (GC–MS), and the odor quality as perceived at the sniffing port during gas chromatography–olfactometry (GC–O) compared to data obtained from authentic reference compounds analyzed under equal conditions. ^2^ Perceived odor quality at the sniffing port during GC–O analysis. ^3^ Fractions were obtained after separating acidic volatiles (AV) and neutral/basic volatiles (NBV). ^4^ Retention index: calculated by linear interpolation from the retention time of the compound and the retention times of adjacent *n*-alkanes. ^5^ Flavor dilution factor: the dilution factor of the highest diluted spirulina volatile isolate in which the odorant was detectable during GC–O. ^6^ The compounds were not separated on the column used for aroma extract dilution analysis (AEDA); the FD factor refers to the mixture. ^7^ An unequivocal mass spectrum could not be obtained from the spirulina volatile extract; identification was based on the remaining criteria stated in footnote 1 and by spiking experiments using the gas chromatography–olfactometry/flame ionization detector (GC–O/FID) system.

## Data Availability

The original contributions presented in this study are included in the article. Further inquiries can be directed to the corresponding author.

## Supplementary Materials

The following supporting information can be downloaded at: https://www.mdpi.com/article/10.3390/ijms26146767/s1. Additional information on GC Instruments. References [55,56] are cited in the Supplementary Materials.

## Abbreviations

The following abbreviations are used in this manuscript:

AEDA	Aroma extract dilution analysis
aSAFE	Automated solvent-assisted flavor evaporation
AV	Acidic volatiles
CI	Chemical ionization
EI	Electron ionization
FD	Flavor dilution
FID	Flame ionization detector
GC	Gas chromatography
GC–GC	Two-dimensional heart-cut gas chromatography
GC×GC	Comprehensive two-dimensional gas chromatography
GC–O	Gas chromatography–olfactometry
KFO	Key food odorant
MS	Mass spectrometry
NBV	Neutral and basic volatiles
NCI	Negative chemical ionization
RI	Retention index
SH	Static headspace
SPME	Solid-phase microextraction
PTV	Programmable temperature vaporizing
TOF	Time-of-flight
