# Supplementary material for: Deciphering Important Odorants in a Spirulina (Arthrospira platensis) Dietary Supplement by Aroma Extract Dilution Analysis Using Offline and Online Fractionation Approaches
Source: Int J Mol Sci. 2025 Jul 15;26(14):6767. doi: 10.3390/ijms26146767 (PMC12296105; doi:10.3390/ijms26146767)
Supplement: Supplementary file 1 [file ijms-26-06767-s001.zip › ijms-3654546-supplementary.pdf]

# – Supplementary Materials –

# Deciphering Important Odorants in a Spirulina (*Arthrospira platensis*) Dietary Supplement by Aroma Extract Dilution Analysis Using Offline and Online Fractionation Approaches

**Aikaterina Paraskevopoulou [1,2,3], Veronika Mall [1], Theodoros M. Triantis [3], Triantafyllos Kaloudis [3,4], Anastasia Hiskia [3], Dimitra Dimotikali [2] and Martin Steinhaus [1,*]**

[1] Leibniz Institute for Food Systems Biology at the Technical University of Munich (Leibniz-LSB@TUM), Freising 85354, Germany; k.paraskevopoulou@inn.demokritos.gr (A.P.); v.mall.leibniz-lsb@tum.de (V.M.); m.steinhaus.leibniz-lsb@tum.de (M.S.)
[2] School of Chemical Engineering, National Technical University of Athens, Athens 15780, Greece; demot@chemeng.ntua.gr (D.D.)
[3] Institute of Nanoscience and Nanotechnology, National Center for Scientific Research "Demokritos", Athens 15341, Greece; t.triantis@inn.demokritos.gr (T.M.T.); kaloudis@eydap.gr (T.K.); a.hiskia@inn.demokritos.gr (A.H.)
[4] AquOmixLab, Department of Water Quality Control, Athens Water Supply and Sewerage Company (EYDAP SA), Athens 11146, Greece
* Correspondence: m.steinhaus.leibniz-lsb@tum.de; Tel.: +49 8161 71 2991

## Additional Information on Gas Chromatography (GC) Instruments

*SM1. Gas Chromatography–Olfactometry/Flame Ionization Detector (GC–O/FID) Instrument*

A Trace gas chromatograph (Thermo Fisher Scientific, Dreieich, Germany) was equipped with a cold-on-column inlet, a flame ionization detector (FID, 250 °C base temperature), and a sniffing port custom-made from aluminum (230 °C base temperature) [55]. The fused silica capillary was either a DB-FFAP column (30 m × 0.32 mm i.d., 0.25 µm film thickness; Agilent Technologies, Waldbronn, Germany) or a DB-5 column (30 m × 0.32 mm i.d., 0.25 µm film thickness; Agilent Technologies). The carrier gas was helium at 70 kPa (DB-FFAP) and 50 kPa (DB-5) constant pressure, resulting in a 2 mL/min flow at 40 °C oven temperature. The injection volume was 1 µL. The initial oven temperature of 40 °C was held for 2 min, followed by a gradient of 6 °C/min until the final temperature of 230 °C (DB-FFAP) or 240 °C (DB-5) was reached. The final temperature values were held for 5 min. A Y-shaped glass splitter connected the end of the column with two uncoated but deactivated fused silica capillaries, each 50 cm × 0.25 mm i.d., which delivered the column effluent in two equal parts to the FID (250 °C base temperature) and the sniffing port (230 °C base temperature), respectively. During the GC–O/FID analysis, a trained assessor placed the nose directly above the sniffing port and evaluated the effluent. Whenever an odor was detected, the position and the odor quality were marked in the FID chromatogram plotted by a recorder SE 120 (Asea Brown Boveri, Zurich, Switzerland). Retention indices (RI) of each odor event were calculated from their retention times and the retention times of adjacent *n*-alkanes by linear interpolation [56].

*SM2. Comprehensive Two-Dimensional Gas Chromatography–Time-of-Flight Mass Spectrometry (GC×GC–TOFMS) Instrument*

A 6890 Plus gas chromatograph (Agilent Technologies) was equipped with a Combi PAL autosampler (CTC Analytics, Zwingen, Switzerland) and a KAS4 inlet (Gerstel, Mülheim/Ruhr, Germany). The fused silica capillary in the first dimension was a DB-FFAP column (30 m × 0.25 mm i.d., 0.25 µm film thickness; Agilent Technologies). The carrier gas was helium at a 2 mL/min constant flow. The injection volume was 2 µL. The initial oven temperature of 40 °C was held for 2 min, followed by a gradient of 6 °C/min until the final temperature of 230 °C. The final temperature value was held for 5 min. The end of the first column was connected via a liquid nitrogen-cooled dual-stage quad-jet modulator (LECO, Mönchengladbach, Germany) to a DB-1701 column (2 m × 0.18 mm i.d., 0.18 µm film thickness; Agilent Technologies) inside the secondary oven, which was mounted inside the primary GC oven. The modulation time was 4 s. The initial temperature of the second oven of 50 °C was held for 2 min, followed by a gradient of 6 °C/min until the final temperature of 250 °C. The final temperature value was held for 5 min. The end of the second column was connected to a Pegasus III TOF mass spectrometer (LECO). The spectrometer was operated in electron ionization (EI) mode at 70 eV with a scan range of *m/z* 35–350 and a scan rate of 100 spectra/s. The data was analyzed using GC Image 2.1b5 (GC Image, Lincoln, NE, USA).

*SM3. Two-Dimensional Heart-Cut Gas Chromatography–Olfactometry/Mass Spectrometry (GC–GC–O/MS) Instrument*

A Trace 1310 gas chromatograph (Thermo Fisher Scientific) was equipped with a TRI Plus RSH autosampler (Thermo Fisher Scientific), a programmable temperature vaporizing (PTV) inlet, an FID (250 °C base temperature), and a sniffing port custom-made from aluminum (230 °C base temperature) [55]. The fused silica capillary was a DB-FFAP column (30 m × 0.25 mm i.d., 0.25 µm film thickness; Agilent Technologies). The carrier gas was helium at a 1 mL/min constant flow. The injection volume was 2 µL. The initial oven temperature of 40 °C was held for 2 min, followed by a gradient of 6 °C/min until the final temperature of 230 °C. The final temperature value was held for 5 min. The end of the first column was connected to a Deans switch (Trajan, Sprockhovel, Germany) used for heart-cutting. The Deans switch directed the eluate of the column via deactivated fused silica capillaries (0.1 mm i.d.) time-programmed

either simultaneously to the FID and the sniffing port used as monitor detectors or to a second fused silica capillary, which was a DB-1701 column (30 m × 0.25 mm i.d., 0.25 µm film thickness; Agilent Technologies). This column was installed in a second Trace 1310 gas chromatograph (Thermo Fisher Scientific). The capillary to the second column first passed through a heated (250 °C) hose connecting the two gas chromatographs and then through a liquid nitrogen-cooled trap to refocus the heart cut. The second oven's initial oven temperature of 40 °C was held for 2 min, followed by a gradient of 6 °C/min until the final temperature of 240 °C. The final temperature value was held for 5 min. The second column's end was linked to a QExactive GC Orbitrap mass spectrometer (Thermo Fisher Scientific). The spectrometer was operated in EI mode at 70 eV with a scan range of *m*/*z* 40–250, in chemical ionization (CI) mode, or in negative chemical ionization (NCI). Both CI modes were applied using isobutane as the reagent gas and a scan range of *m*/*z* 90–250. The data was analyzed using Xcalibur 4.1.31.9 (Thermo Fisher Scientific) and NIST library 2.3 (2017; NIST, Gaithersburg, MD, USA).

*SM4. Static Headspace–Gas Chromatography–Olfactometry/Mass Spectrometry (SH–GC–O/MS) Instrument*

A Trace Gas Chromatograph Ultra (Thermo Fisher Scientific) was equipped with a Combi PAL autosampler (CTC Analytics), a cold-on-column inlet, a custom-made air purge, a Cold Trap 915 (Thermo Fisher Scientific), an FID (250 °C base temperature), and a sniffing port custom-made from aluminum (230 °C base temperature) [55]. The fused silica capillary was a DB-5 column (30 m × 0.25 mm i.d., 1.0 µm film thickness; Agilent Technologies). The carrier gas was helium at 100 kPa constant pressure, resulting in a 2 mL/min flow at 40 °C oven temperature. The cold trap was initially cooled to –150 °C to trap injected volatiles on a deactivated fused silica capillary (0.2 m × 0.53 mm i.d). Headspace volumes of 125 µL–10 mL were injected using a gas-tight syringe while purging the air from the cooled trap. After the injection, the cold trap was heated to 250 °C to transfer the cryofocused compounds to the DB-5 column. The initial oven temperature of 0 °C was achieved using liquid nitrogen oven cooling and was held for 2 min, followed by a gradient of 6 °C/min until 50 °C and a gradient of 40 °C/min until the final temperature of 240 °C. The final temperature value was held for 5 min. The end of the column was connected to two Y-shaped glass splitters in series, and the column effluent was divided into three parts using deactivated fused silica capillary pieces. The first part was transferred to the FID, the second was conveyed to the sniffing port used as a monitor detector, and the third was directed to a Paul ion trap mass spectrometer Saturn 2200 (Agilent Technologies). The spectrometer was operated in EI mode at 70 eV with a scan range of *m*/*z* 40–250 or CI mode using methanol as reagent gas and a scan range of *m*/*z* 55–250. The data was analyzed using MS Workstation 6.9.1 (Agilent Technologies).
